# Exploratory unsupervised machine learning of angiogenesis biomarkers in a phase II advanced cervical cancer trial of radiochemotherapy with or without neoadjuvant chemotherapy

**DOI:** 10.1016/j.clinsp.2025.100723

**Published:** 2025-07-30

**Authors:** Roberto J. Arai, Thiago R. da Costa, Renata Colombo Bonadio, Silvaneide Ferreira, Laura Sichero, Hugo P. Monteiro, Arnold Stern, Maria Del Pilar Estevez-Diz

**Affiliations:** a Instituto do Câncer do Estado de São Paulo (ICESP), Faculdade de Medicina da Universidade de São Paulo, São Paulo, SP, Brazil; b AW Scientific Engineering, Londrina, PR, Brazil; c Instituto D’Or de Pesquisa e Ensino (IDOR), São Paulo, SP, Brazil; d Comprehensive Center for Precision Oncology, Universidade de São Paulo, São Paulo, SP, Brazil; e Department of Biochemistry, Center of Cellular and Molecular Therapy, Escola Paulista de Medicina, Universidade Federal de São Paulo, São Paulo, SP, Brazil; f Grossman School of Medicine, New York University, NY USA

**Keywords:** Angiogenesis, Biomarkers, Cervical cancer, Neoadjuvant therapy, Chemoradiotherapy

## Abstract

**Objective:**

In a prospective, randomized phase II study, exploratory data analysis was conducted to evaluate angiogenesis-associated plasma protein levels in patients with locally advanced cervical cancer

**Methods:**

Participants were divided into two groups: Group A received neoadjuvant cisplatin and gemcitabine treatment (NAC) followed by chemoradiation with cisplatin and brachytherapy (CRT), while Group B received only CRT. Plasma samples were collected from patients in Group A at three time points: baseline, after NAC, and after CRT. Group B patients had samples taken at two time points: baseline and after CRT. The study analyzed an angiogenesis-associated panel of plasma proteins, including angiopoietin-2, G-CSF, endothelin-1, FGF-1, FGF-2, follistatin, IL-8, HGF, EGF, HB-EGF, PLGF, VEGF-A, VEGF-C, and VEGF- D. Receiver Operating Characteristic (ROC) analyses assessed the predictive value of baseline biomarkers for 12, 24, and 36-month survival outcomes. Additionally, Principal Component Analysis (PCA) was applied to post-CRT biomarker changes to identify coordinated modulation patterns. PCA was based on normalized delta values and eigenvector loadings, enabling identification of biomarkers aligned with Progression-Free Survival (PFS) and Overall Survival (OS).

**Results:**

Significant differences were observed in the levels of HB-EGF, IL-8, PLGF, and VEGF-C between Groups A and B following CRT. Additionally, angiopoietin-2 levels showed a significant increase in Group B only. NAC treatment in Group A appeared to downregulate IL-8. CRT induced significant changes in HB-EGF, IL-8, PLGF, and VEGF-C levels in both groups. Patients in Group B demonstrated improved PFS and OS compared to those in Group A. Despite these differences in survival outcomes, the authors observed no significant intergroup differences in the tested biomarkers after completion of CRT. ROC analysis of baseline angiogenesis biomarkers demonstrated limited predictive sensitivity for survival outcomes. However, PCA of biomarker changes following CRT highlighted VEGF-A, HB-EGF, and angiopoietin-2 as variables associated with PFS and OS.

**Conclusion:**

Baseline biomarker levels were not predictive of long-term outcomes. In contrast, CRT alone modulated key angiogenic biomarkers, and post-CRT biomarker changes were associated with improved survival. Such biomarker alterations were not observed following NAC, which was not associated with clinical benefit in this study. These findings underscore the value of dynamic biomarker evaluation and highlight how treatment strategies differentially impact biomarker profiles in advanced cervical cancer.

## Highlights

• Baseline angiogenic biomarkers showed a weak predictive value by the ROC curve.
• CRT changes VEGF-A, HB-EGF, angiopoietin-2, and HGF.
• These alterations are not observed after NAC.
• Biomarker patterns post-CRT were associated with improved outcomes by PCA.
• ML may aid future stratification of angiogenesis profiles.

## Introduction

The results of the GOG240 showed that combining angiogenesis inhibition with chemotherapy improved the median overall survival by 3.7 months in patients with recurrent, persistent, or metastatic cervical cancer. The benefits were particularly notable in patients with squamous cell carcinoma who had previously received platinum-based chemotherapy and radiation therapy, and those who were experiencing recurrent or persistent disease^1^.

Angiogenesis involves multiple signaling events that activate resting endothelial and stromal cells, which then acquire the ability to remodel the extracellular matrix, proliferate, differentiate, and stabilize as blood vessels via Nitric Oxide (NO) mediation^2^, ^3^, ^4^, ^5^. Various pro-angiogenic factors are known to be involved in this process, including Vascular Endothelial Growth Factor (VEGF), Platelet-Derived Growth Factor (PDGF), Hepatocyte Growth Factor (HGF), basic Fibroblast Growth Factor (bFGF), Matrix Metalloproteinases (MMPs), Transforming Growth Factor (TGF)-β, Interleukin (IL)-8 and angiopoietins^6^. In breast cancer, neoadjuvant chemotherapy has been shown to modulate angiogenesis-associated proteins, such as VEGF and FGF, potentially enhancing the anti-tumor effects of subsequent treatments^7^.

The angiogenesis pathway, mediated by VEGF and its tyrosine kinase receptors (VEGFR-1 and VEGFR-2)^8^, has been extensively studied as a potential predictive factor for cancer treatment outcomes. However, conflicting evidence suggests that targeting this pathway alone may not serve as a universal therapeutic strategy^9^. To better guide treatment selection, incorporating a broader panel of plasma proteins could provide valuable insights^10^. Further studies are needed to identify potential responders who may benefit from therapies that regulate angiogenesis as part of their anti-tumor mechanism.

This study aimed to determine whether angiogenic marker protein levels in plasma samples could provide insights through exploratory analysis into treatment efficacy for selected patients with advanced cervical carcinoma.

## Materials and methods

### Study design, participants, and sample collection

Samples were collected from patients participating in a two-arm, randomized, open-label, phase II trial conducted in a single academic cancer center, the Instituto do Cancer do Estado de São Paulo (NCT01973101), in São Paulo, Brazil. The study included 110 patients. Samples were collected from the first 80 patients. Eligible patients were required to have histological confirmation of local advanced cervical carcinoma stages IIB to IVA. Tumor staging was assessed according to the staging system of the International Federation of Gynecology and Obstetrics (FIGO)^11^. Group A received Neoadjuvant Chemotherapy (NAC) followed by Chemoradiotherapy and Brachytherapy (CRT), while Group B received only CRT. NAC consisted of intravenous cisplatin 50 mg/m^2^ on day 1 and gemcitabine 1000 mg/m^2^ on day 1 and day 8 every 3 weeks for three cycles. NAC was followed by standard CRT, which started 3- to 4-weeks after the last cycle of NAC. The control group received standard CRT only. Standard CRT consisted of weekly cisplatin 40 mg/m2 for 6 weeks concurrent with radiotherapy. Pelvic external beam radiation therapy was delivered at 45 to 50.4 Gy in 25 to 28 fractions. For all patients with adequate geometry for intracavitary brachytherapy, this was delivered in four weekly fractions of 7 or 7.5 Gy (total dose of 28 or 30 Gy). If unsuitable for brachytherapy, patients receive an external beam boost of up to 14 Gy for all gross tumor diseases. For patients eligible for brachytherapy who had nodal disease or extensive parametrial disease, a boost dose of 10 to 14 Gy was allowed.

### Sample collection and plasma angiogenesis biomarkers assessments

Plasma samples from patients in group A were collected at three different time points (at baseline (*n* = 34), after NAC (*n* = 24), and after CRT (*n* = 27). Samples from group B were collected at two different time points at baseline (*n* = 40) and after CRT (*n* = 34). All patients were randomized and assigned 1:1 to NAC with cisplatin and gemcitabine, followed by standard CRT or standard CRT alone. Samples were collected according to institutional Standard Operational Procedures. Briefly, plasma samples were collected in EDTA-containing tubes. Samples were incubated for 30 min at room temperature and centrifuged at 1500 g for 10 min at 4 °C. Aliquots were then frozen at −80 °C until testing. Non-viable samples, such as those with hemolysis or insufficient volume, were excluded from the analysis. Some patients did not have viable baseline samples due to processing issues, leading to slight differences in final sample counts. Plasma levels of angiopoietin-2, G-CSF, endothelin-1, FGF-1, FGF-2, follistatin, IL-8, HGF, EGF, HB-EGF, PLGF, VEGF-A, VEGF-C, and VEGF-D were assessed by a Multiplex Assay using the Luminex® technology. The immunoassay Milliplex® was conducted according to the manufacturer’s protocol. Although 110 patients were enrolled, biomarker profiling was restricted to the first 80 participants due to limitations in available assay reagents and quality-controlled sample material. This decision was made to ensure analytical consistency, avoid inter-batch variability, and maintain rigorous assay performance. As a result, the final analytical cohort was defined by logistical feasibility rather than clinical characteristics. While this may limit the statistical power and generalizability of the findings, it ensured methodological reliability and homogeneity in biomarker testing conditions.

### Statistical analysis

Statistical analysis was performed with the software SPSS 20.0 (SPSS, Inc., Chicago, IL, USA). Samples were grouped and evaluated with a non-parametric Mann-Whitney test, in which the authors considered the points as independent groups. The Friedman test (ANOVA) was used to analyze group A (with three evaluation points). Interquartile statistics were used to show the results. Missing data were considered completely at random (MCAR), and patients without at least one post-baseline collection were excluded. Overall Survival (OS) and Progression-Free Survival (PFS) were analyzed using the Kaplan-Meier method, and the log-rank test performed comparisons between groups. All tests were two-sided, and statistical significance was defined as a p-value of ≤0.05.

### Receiver operating characteristic (ROC)

The role of baseline angiogenesis-related biomarkers was explored in predicting clinical outcomes. We performed Receiver Operating Characteristic (ROC) curve analyses^12^ using plasma concentrations of 14 biomarkers measured before treatment initiation. The biomarkers assessed included: EGF, angiopoietin-2, G-CSF, endothelin-1, FGF-1, follistatin, IL-8, HGF, HB-EGF, PLGF, VEGF-C, VEGF-D, FGF-2, and VEGF-A. For each patient, binary outcome variables were generated for OS and PFS at 12-, 24-, and 36-months. Patients who experienced the event of interest (death for OS, or progression/death for PFS) before each respective timepoint were coded as 1 (event), while those who remained alive and/or progression-free beyond the timepoint were coded as 0 (non-event). The ROC curve was generated by plotting the true positive rate (sensitivity) against the false positive rate at varying biomarker thresholds. An AUC of 1.0 indicates perfect discrimination, whereas an AUC of 0.5 indicates performance no better than random chance. All analyses were performed using Python (v3.11). Before calculation, missing biomarker values were excluded on a per-variable basis to maintain consistency across comparisons. ROC analyses were conducted separately for each biomarker and each timepoint, and only timepoints with sufficient class balance (i.e., at least one event and one non-event) were included in the final AUC evaluation.

### Principal component analysis (PCA) based on eigenvectors to explore biomarker modulation following CRT

PCA was performed to analyze multidimensional changes in angiogenesis biomarkers from baseline to post-CRT. The input data consisted of delta values (post-CRT minus baseline levels, adjusted by baseline levels) for 14 plasma biomarkers: angiopoietin-2, G-CSF, endothelin-1, FGF-1, FGF-2, follistatin, IL-8, HGF, EGF, HB-EGF, PLGF, VEGF-A, VEGF-C, and VEGF-D. Only patients with complete paired samples were included. The dataset was normalized by mean centering and scaling. PCA was then performed using the covariance matrix, resulting in a set of eigenvectors, each representing a linear combination of original biomarkers that defines a principal component^13^. The corresponding eigenvalues indicate the amount of variance each component explains. The first two components, PC1 and PC2, which accounted for 23.5 % and 17.5 % of the total variance, respectively, were selected for further interpretation. A PCA loading plot was generated to visualize the orientation and magnitude of the eigenvectors: The direction of each vector represents the correlation between biomarkers. Parallel vectors indicate positive correlation, opposite vectors indicate inverse relationships, and orthogonal vectors imply statistical independence. Length reflects the strength of each biomarker’s contribution to PC1 and PC2. Color coding was applied to show the relative contribution of each biomarker to the total variance explained.

### STARD guidelines

This study was conducted following the Standards for Reporting of Diagnostic Accuracy Studies (STARD) 2015 guidelines^14^.

### Ethical considerations and standards for human research

This study was reviewed and approved by the institutional Ethics Committee under the approval code of *Certificado de Apresentação de Apreciação Ética* (CAAE) 01.848.412.5.0000.0065. This work was conducted according to the Code of Ethics of the World Medical Association, the Declaration of Helsinki, local regulations, and Good Clinical Practice. The informed consent was obtained according to local and international legislation and standards. All privacy rights were granted.

### Presentation

This study was previously presented by RJA at the AACR-NCI-EORTC International Conference on Molecular Targets and Cancer held in Boston, USA, in October 2019. DOI: 10.1158/1535–7163.TARG-19-A038.

## Results

### Patient population

**Fig. 1:**
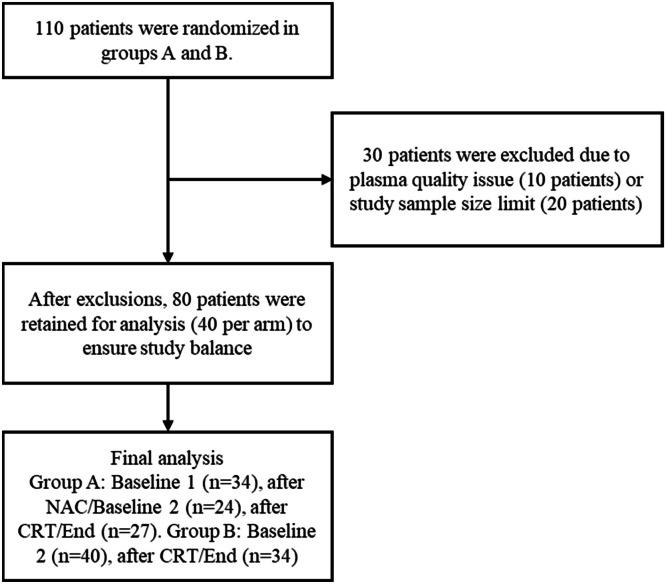
STARD flow diagram illustrates the patient selection, exclusion, and sample collection process for the study. Samples were collected from 80 patients (balanced 40 patients per arm), following exclusions based on plasma quality and study size limits.

A total of 110 patients were initially randomized into two study groups. Following enrollment, 30 patients were excluded from the study: 10 due to poor plasma quality and 20 due to study sample size limitations (Fig. 1). This exclusion process ensured the integrity and feasibility of the analysis. After exclusions, a final cohort of 80 patients (40 per group) was retained for downstream analysis to maintain balance between the study's arms. Patients consented to participate and authorized blood collection (40 patients in group A and 40 in group B). Most patients were diagnosed with squamous cell carcinoma: 90.0 % in group A and 85.0 % in group B. Patients had stage IIB disease in 47.9 % of the cases in both groups, and mostly ECOG 1 (Eastern Cooperative Oncology Group) (55.0 % in group A and 52.5 in group B). Lymph nodes were negative in 60.0 % of patients from group A, and 62.5 % in group B (Table 1).

**Table 1:** Patient demographics and baseline clinical characteristics of the modified intention-to-treat population.

	N° of patients ( %)	
Characteristic	NAC (*n* = 40)	CRT (*n* = 40)
Age (median)	48.9	45.55
Range	(24.7‒69.7)	(20.0‒67.7)
ECOG		
0	18 (45.0)	18 (45)
1	22 (55.0)	21 (52.5)
2	‒	1 (2.5)
Histology		
Squamous Cell Carcinoma	36 (90.0)	34 (85.0)
Adenocarcinoma	4 (10.0)	6 (15.0)
Stage		
IIB	19 (47.5)	19 (47.5)
IIIA	‒	1 (2.5)
IIIB	18 (45.0)	14 (35.0)
IVA	3 (7.5)	6 (15.0)
Lymph node		
Positive	16 (40.0)	15 (37.5)
Negative	24 (60.0)	25 (62.5)

### Longitudinal assessment of 14 circulating proteins reveals distinct biomarker changes after CRT

**Fig. 2:**
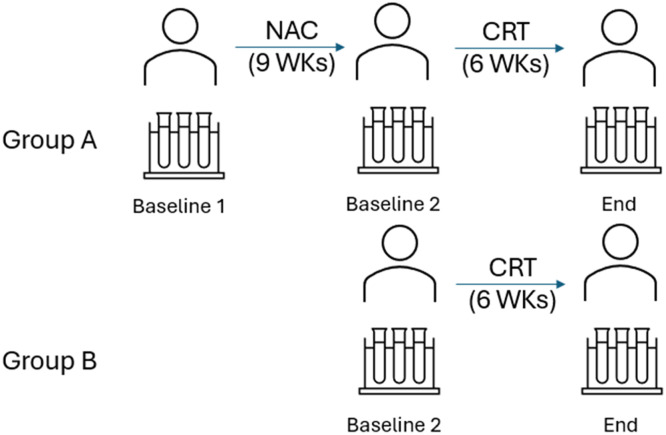
Sample collection scheme and time points. Samples were collected within seven days before NAC (baseline 1) or CRT (baseline 2) and 7 days after CRT (end).

**Fig. 3:**
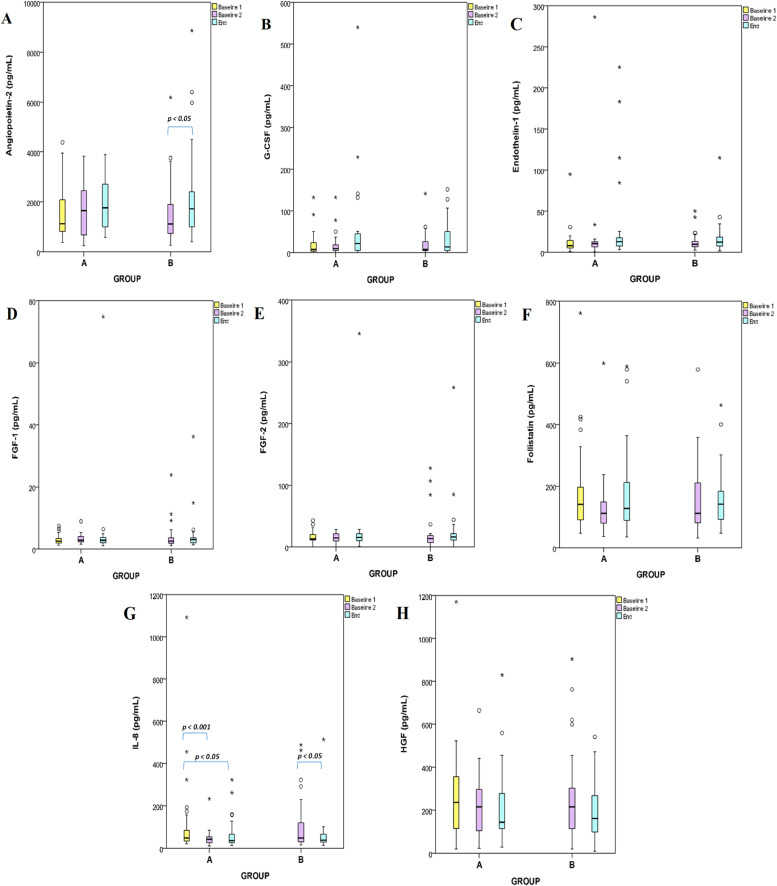

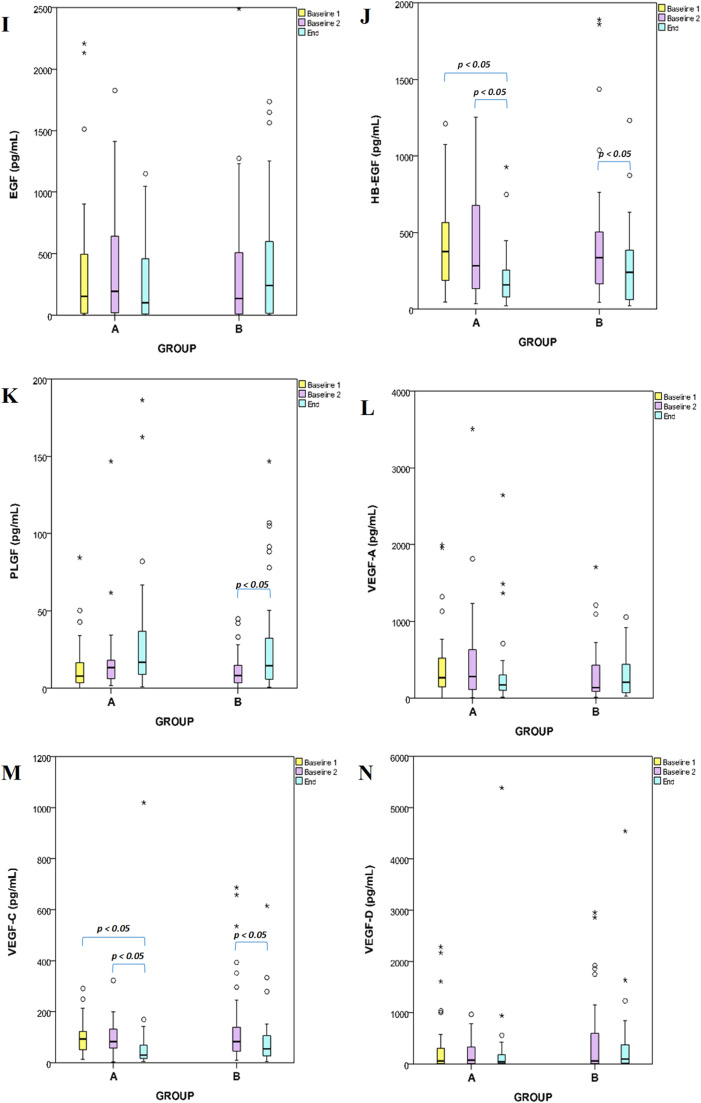
Box plots comparing group A and B of angiogenesis plasma biomarkers collected before NAC (baseline 1) or CRT (baseline 2) and after CRT (end). Figure (A) angiopoietin-2, (B) G-CSF, (C) endothelin-1, (D) FGF-1, (E) FGF-2, (F) follistatin, (G) IL-8, (H) HGF, (I) EGF, (J) HB-EGF, (K) PLGF, (L) VEGF-A, (M) VEGF-C, and (N) VEGF-D. The middle line represents the median; the lower hinge corresponds to the first quartile (25th); the upper hinge corresponds to the third quartile (75th); upper and lower whiskers extend from the hinge, respectively to the largest value and smallest value data beyond the whiskers are outliers (circles) and extremes (asterisks); p-values (*p* < 0.05 and *p* < 0.001) are indicated in the figures.

The circulating levels of the 14 proteins were assessed in plasma samples collected from patients included in both groups. Angiopoietin-2, G-CSF, endothelin-1, FGF-1, follistatin, IL-8, HGF, EGF, HB-EGF, PLGF, VEGF-D, VEGF-A, VEGF-C, and FGF-2 were determined by Multiplex technology. To explore treatment-specific effects, each biomarker was analyzed both within and between study groups with the Mann-Whitney approach. Intergroup comparisons were performed at matched timepoints to assess differential biomarker modulation by NAC and CRT. In Group A (NAC+CRT), biomarker levels were evaluated at baseline, post-NAC, and post-CRT, while in Group B (CRT only), levels were compared between baseline and post-CRT. This approach enabled the identification of distinct modulation patterns attributable to neoadjuvant chemotherapy and chemoradiotherapy, and highlighted differences in biomarker trajectories across treatment arms (Fig. 2). The authors found that plasma levels of angiopoietin-2 and PLGF significantly increased in group B (after CRT), whereas HB-EGF and VEGF-C significantly decreased in groups A and B after CRT. Specifically, IL-8 decreased significantly after NAC (*p* < 0.01) treatment and in group B after CRT (*p* < 0.05). The protein levels of EGF, G-CSF, endothelin-1, FGF-1, FGF-2, follistatin, HGF, and VEGF-D showed no significant alterations (Fig. 3).

### Kaplan-Meier analysis of OS and PFS outcomes and plasma biomarker modulation following CRT

**Fig. 4:**
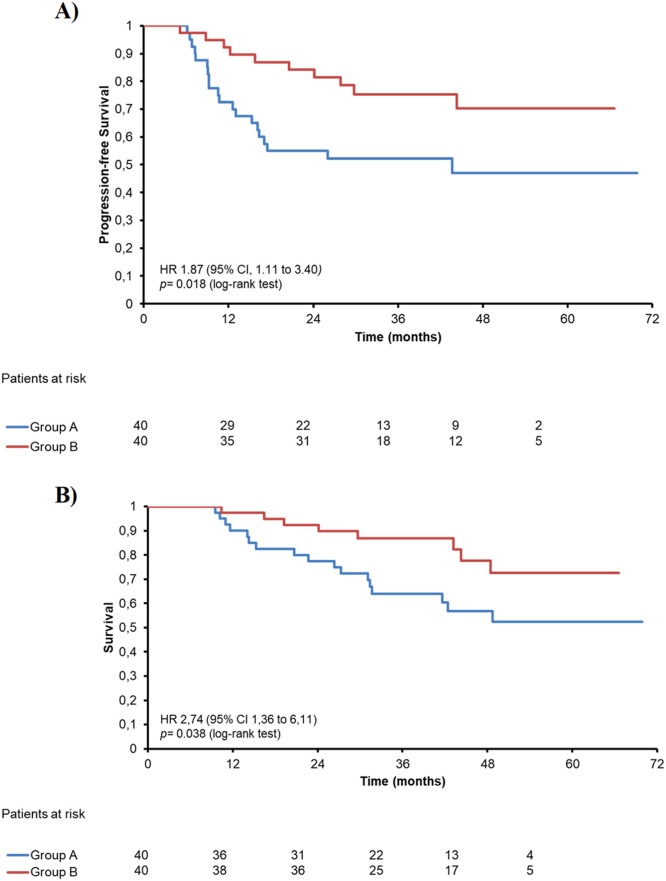
Kaplan-Meyer curves of patients receiving NAC and CRT (group A, *n* = 40) or CRT only (group B, *n* = 40). Kaplan-Meier curves for (A) progression-free survival (PFS) and (B) overall survival (OS).

The authors explored the association of angiogenesis markers with PFS and OS using Kaplan-Meier analysis, which was carried out in both groups. Patients receiving NAC had significantly lower PFS (log-rank *p* = 0.018; Fig. 4A) and OS (log-rank *p* = 0.038; Fig. 4B) compared to CRT alone. Nevertheless, no association of any studied biomarkers was found in patients receiving or not receiving NAC with PFS or OS. Limited predictive value of baseline angiogenesis biomarkers by ROC analysis.

The ROC analysis showed that baseline biomarkers demonstrated limited predictive value for long-term survival outcomes (Table 2). For OS at 12-months, EGF had the highest (AUC = 0.63), while Endothelin-1 was the top performer for PFS at the same timepoint (AUC = 0.62). However, at the 24- and 36-months, AUC values generally declined or remained close to 0.5, indicating weak prognostic power. At 36-months, angiopoietin-2 showed moderate predictive ability for PFS (AUC = 0.62), whereas no biomarker exceeded an AUC of 0.55 for OS.

**Table 2:** AUC values from receiver operating characteristic (ROC) curve analyses evaluating the predictive performance of angiogenesis plasma biomarkers for overall survival (OS) and progression-free survival (PFS) at 12-, 24-, and 36-months. Biomarker levels were assessed at baseline 2 before CRT initiation. AUC values closer to 1.0 indicate better predictive ability, whereas values near 0.5 suggest limited or no discriminatory power. The last row indicates the number of available events and corresponding biomarker measurements for each time point.

	OS			PFS		
Months	12	24	36	12	24	36
EGF	0.63	0.48	0.32	0.45	0.42	0.39
Angiopoietin-2	0.51	0.42	0.51	0.48	0.41	0.62
Endothelin-1	0.61	0.42	0.48	0.62	0.45	0.49
FGF-1	0.38	0.39	0.35	0.34	0.4	0.34
FGF-2	0.43	0.34	0.47	0.39	0.37	0.53
Follistatin	0.68	0.51	0.58	0.63	0.55	0.62
G-CSF	0.51	0.41	0.44	0.54	0.44	0.56
HB-EGF	0.51	0.39	0.43	0.46	0.4	0.47
HGF	0.36	0.37	0.48	0.39	0.43	0.62
IL-8	0.37	0.4	0.46	0.43	0.45	0.55
PLGF	0.38	0.24	0.26	0.37	0.3	0.34
VEGF-A	0.29	0.32	0.44	0.35	0.38	0.49
VEGF-C	0.62	0.5	0.6	0.56	0.54	0.55
VEGF-D	0.57	0.55	0.39	0.47	0.56	0.51
**Number of events/Total**	14/64	16/64	21/64	6/64	19/64	20/64

### Exploratory unsupervised machine learning analysis of biomarker dynamics

**Fig. 5:**
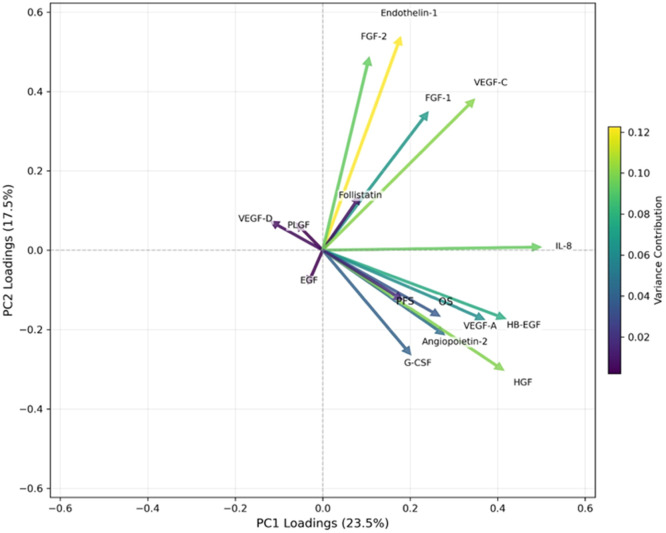
Principal component analysis (PCA) was performed to explore the relationships between changes in biomarker levels following CRT and their association with overall data variance. In this plot, the values represent the difference between baseline and post-CRT levels for each biomarker. The color of each vector represents the contribution of these biomarker changes to the total variance (as shown in the color bar). The length of the vector indicates its influence on the principal components. The direction of the vectors illustrates the correlations between the changes in biomarker levels, where vectors pointing in similar directions suggest positive correlations, opposing directions indicate negative correlations, and orthogonal (perpendicular) vectors suggest minimal or no linear correlation.

To explore patterns of coordinated biomarker modulation induced by CRT, the authors applied Principal Component Analysis (PCA) to the differences in plasma biomarker levels between baseline and post-CRT for each patient. This unsupervised multivariate approach allowed visualization of the dataset's variance structure and inter-marker relationships. The first two principal components (PC1 and PC2) accounted for 23.5 % and 17.5 % of the total variance, respectively. The loading plot (Fig. 5) displays the direction and magnitude of each biomarker’s contribution to these components. VEGF-A, HB-EGF, HGF, and angiopoietin-2 exhibit vector directions of OS and PFS, indicating positive correlations with survival. These biomarkers may contribute to better clinical outcomes when modulated favorably by CRT. G-CSF also aligns moderately with survival vectors, suggesting its role in therapy response. In contrast, biomarkers such as IL-8, FGF-1, FGF-2, and Endothelin-1 are oriented in the opposite direction, potentially indicating inverse correlations with survival, i.e., increased levels may be associated with worse outcomes. Orthogonal vectors such as VEGF-D, EGF, and PLGF indicate a weak or negligible linear relationship with OS or PFS in this multivariate space. The loading magnitudes (indicated by arrow length and color gradient) show that VEGF-C, FGF-2, and Endothelin-1 contribute most strongly to variance, suggesting that their changes are prominent across the dataset but may not be positively aligned with survival endpoints.

These findings highlight a distinct multivariate signature among CRT-modulated biomarkers that parallels clinical benefits. The alignment of survival outcomes with angiopoietin-2 and VEGF family members underscores their potential as prognostic indicators, justifying future validation through supervised learning and longitudinal modeling.

## Discussion

In cervical cancer, higher tumor vascularity has been associated with lower overall survival rates in patients who received pelvic irradiation after loco-regional control^6^. Reports have confirmed that cervical carcinomas with overexpression of CD31, a marker that indicates tumor angiogenic activity, and increased microvessel density are correlated with worse survival^15^. A prognostic factor is indicative of the biology of the tumor and connects with the patient’s prognosis, despite the therapy employed. Predictive factors provide information on whether the patient will benefit from treatment^16^. For instance, the ECOG4599 study demonstrated significant survival benefits when adding bevacizumab to paclitaxel-carboplatin in Non-Small Cell Lung Cancer (NSCLC). Notably, VEGF-C protein expression was demonstrated as a prognostic factor in NSCLC, and the predictive value of increased response to anti-VEGF therapy was shown^17^.

Pharmacodynamic markers suggest that the drug effects may not accurately reflect patients' clinical status. In contrast, modulation of a surrogate marker shows a stronger correlation with clinical outcomes. The present findings reveal that both groups negatively modulated plasma angiogenesis proteins after CRT, such as HB-EGF and VEGF-C. However, angiopoietin-2 and PLGF levels were significantly increased post-CRT in group B^18^.

In the present study, we found no significant differences in the levels of biomarkers between groups A and B after CRT. We should acknowledge limitations in the present study, such as the absence of plasma level assessments after objective response, as Gao M et al. demonstrated in patients with esophageal cancer treated with checkpoint inhibitors^12^. These findings indicate that the CRT regimen significantly reduced pro-angiogenic factors of the VEGF family, specifically VEGF-C and PLGF, while levels of angiopoietin-2 and HB-EGF showed an increase. Angiogenesis is regulated by a complex balance between proangiogenic and antiangiogenic factors, maintaining physiological homeostasis. However, in neoplastic tissues, this balance shifts due to the upregulation of proangiogenic factors such as VEGF, FGF-1, PDGF, and angiopoietins, alongside the downregulation of antiangiogenic factors such as thrombospondin, angiostatin, and endostatin. Depending on the context, this dynamic shift, known as the “angiogenic switch”, facilitates or inhibits neovascularization.

In the present study, CRT modulated angiogenesis profiles through signaling pathways involving VEGF-C, HB-EGF, and IL-8. The continued increase in plasma levels of angiopoietin-2 and PLGF suggests that CRT may not significantly affect these proteins. However, the lack of data comparing angiogenesis protein levels in untreated patients prevents us from assessing the direct impact of these markers on disease progression.

Several pathways are involved, including, but not limited to, PI3-kinase and MAPK signaling pathways^19^, ^20^, ^21^, ^22^ to halt tumor vascularization, tumor response, and possibly clinical benefits. Angiopoietins (1 and 2) may have a complementary role in angiogenesis. Angiopoitein-1 stabilizes mature vessels, while angiopoietin-2 is associated with vascular remodeling^23^^,^^24^. The blood vessel angiopoietin-1 destabilization seems critical, but has limited action alone and should act in concert with other pro-angiogenic factors. The combination of angiogenesis markers such as PLGF and VEGF correlates with response in patients previously treated with bevacizumab in the Velour trial^25^. Specifically, plasma PLGF correlates with treatment response to FOLFIRI (fluorouracil, leucovorin, and irinotecan) plus bevacizumab/aflibercept in colorectal cancer. PLGF negatively modulates tumor angiogenesis and sensitizes anti-VEGF antitumoral agents^26^. Despite decades of investigation of angiogenesis as an effective target in cancer therapy, potential responders depend on the type of treatment, coordinated players beyond angiogenic factors, and the patient's clinical status. Currently, no isolated angiogenic biomarker is used in clinical practice.

The unsupervised PCA analysis offered interesting insights into biomarker behavior by capturing coordinated modulation patterns not evident in univariate tests. Importantly, principal components reflecting changes in VEGF-A, HB-EGF, angiopoietin-2, and HGF were positively aligned with OS and PFS, suggesting that CRT’s clinical efficacy may be partially mediated through modulation of these angiogenic pathways. This dynamic clustering highlights a composite signature that warrants further validation as a multivariate surrogate of CRT benefit. The PCA analysis provides a useful overview of this study's complex interplay between angiogenesis biomarkers. The data hints that the effectiveness of CRT in enhancing survival could be related to its potential to decrease levels of pro-angiogenic biomarkers like VEGF-A, EGF, HGF, angiopoietin-2, and perhaps, G-CSF. Collectively, these findings suggest that dynamic shifts in angiogenesis biomarkers following treatment, not static baseline values, are more informative of therapeutic impact. This distinction is critical for future trial designs aiming to incorporate biomarker-guided decision-making. It also opens avenues for integrating temporal biomarker trends with clinical endpoints using supervised learning frameworks in subsequent studies.

Traditional univariate analyses evaluate biomarkers independently and may overlook complex interactions. In contrast, unsupervised multivariate methods like PCA capture coordinated changes across multiple variables, revealing latent patterns not detectable through standard statistics. In the present study, PCA identified biomarker shifts that aligned with survival outcomes, highlighting the added value of this approach in uncovering biologically meaningful signatures beyond individual marker effects.

The previous report demonstrated that NAC with cisplatin and gemcitabine was associated with worse PFS and OS^18^. In contrast, findings from the INTERLACE trial suggest significant improvements in both PFS and OS following NAC. In that trial, a shorter neoadjuvant period, six weeks of weekly carboplatin and paclitaxel, was associated with clinical benefit^27^. The CIRCE trial, on the other hand, employed a longer neoadjuvant regimen consisting of three cycles of cisplatin and gemcitabine administered every 21-days. It remains to be elucidated whether differences in chemotherapy regimens influence the pharmacodynamics and biomarker dynamics in cervical cancer, potentially contributing to these contrasting outcomes.

The initial management of locally advanced cervical cancer as a continuum of care can have profound implications on subsequent treatment opportunities. Based on the results of the KEYNOTE-A18 trial, immunotherapy becomes more integrated into cervical cancer care, being indicated during and after CRT^28^. Emerging evidence highlights a functional interplay between angiogenesis and immune evasion mechanisms. Tumor-derived angiogenic factors, particularly VEGF-A, PLGF, and angiopoietin-2, contribute not only to aberrant vascularization but also to immune suppression by promoting regulatory T-cells (Tregs), impairing dendritic cell maturation, and reducing T-cell infiltration^29^^,^^30^. The PD-1/PD-L1 axis, which inhibits T-cell effector function in the tumor microenvironment^31^, can be exacerbated by angiogenic signaling. Consequently, an immunosuppressive angiogenic milieu may blunt the efficacy of immune checkpoint blockade^30^^,^^32^.

Conversely, modulation of angiogenic pathways by chemoradiotherapy, as observed in the present study through reductions in VEGF-C and HB-EGF and PCA-driven clustering of survival-associated profiles, may reprogram the tumor microenvironment into a more immune-permissive state. This vascular normalization effect can enhance cytotoxic T-cell infiltration and improve the therapeutic index of PD-1–based treatments^33^^,^^34^.

Ultimately, this supports a biomarker-guided, longitudinal treatment framework in which angiogenesis profiling informs not only prognosis but also the selection and sequencing of angiogenesis and immunotherapeutic strategies, aligning with the principles of precision oncology.

## Conclusion

This randomized phase II study demonstrates that baseline plasma levels of angiogenesis-related biomarkers have limited predictive value for survival outcomes in patients with advanced cervical carcinoma, as indicated by low AUC values in ROC analysis. In contrast, dynamic changes in biomarker levels following CRT revealed meaningful biological patterns. CRT alone significantly modulated key angiogenic proteins such as HB-EGF, VEGF-C, IL-8, and PLGF, particularly in the group not receiving NAC. The addition of NAC failed to produce consistent or beneficial modulation of angiogenic markers and was not associated with improved clinical outcomes.

## Study limitations

This study has limitations. First, the sample size was modest, limiting statistical power for subgroup analyses and potentially underestimating biomarker-survival associations. Second, plasma samples were collected at standardized post-treatment intervals for all patients, and not after objective response assessment, which restricts longitudinal interpretation of biomarker dynamics. Third, while PCA revealed relevant biomarker patterns, this unsupervised method does not account for clinical covariates or confounding factors. Additionally, the exploratory nature of the analyses, including ROC evaluations and PCA, precludes definitive conclusions about causality or predictive utility. Finally, the study population was limited to a single academic center, which may affect the generalizability of the findings to broader or more diverse populations.

Unsupervised multivariate analysis using PCA identified coordinated biomarker shifts, particularly in VEGF-A, HB-EGF, HGF, and angiopoietin-2, that positively aligned with survival endpoints, suggesting their relevance as dynamic indicators of treatment response. These findings underscore the potential value of post-treatment biomarker profiling over baseline measurements and highlight CRT’s role as a biologically and clinically effective strategy. Future studies should validate these exploratory insights in larger cohorts and investigate their integration into personalized treatment algorithms.

## Funding

This study was funded by Fundaçao de Amparo a Pesquisa do Estado de São Paulo (FAPESP) Grant n. 14/01696-1 to RJA.

## CRediT authorship contribution statement

**Roberto J. Arai:** Conceptualization, Methodology, Formal analysis, Supervision, Writing – original draft, Funding acquisition. **Thiago R. da Costa:** Formal analysis, Data curation. **Renata Colombo Bonadio:** Writing – review & editing. **Silvaneide Ferreira:** Investigation, Resources. **Laura Sichero:** Writing – review & editing. **Hugo P. Monteiro:** Writing – review & editing. **Arnold Stern:** Writing – review & editing. **Maria Del Pilar Estevez-Diz:** Project administration, Supervision.

## Declaration of competing interest

The authors declare no conflicts of interest.
